# Practical implementation and impact of the 4R principles in ethnopharmacology: Pursuing a more humane approach to research

**DOI:** 10.3389/fphar.2025.1543316

**Published:** 2025-03-28

**Authors:** Jimin Liu, Xiang Zai, Xiaqing Tian, Jiaxuan Li, Shipeng Yan, Taiyi Wang

**Affiliations:** ^1^ Innovation Research Institute of Chinese Medicine, Shandong University of Traditional Chinese Medicine, Jinan, China; ^2^ College of Animal Science and Veterinary Medicine, Shandong Agricultural University, Tai’an, China; ^3^ College of Pharmacy, Shandong University of Traditional Chinese Medicine, Jinan, China; ^4^ Institute of Acupuncture and Moxibustion, Shandong University of Traditional Chinese Medicine, Jinan, China; ^5^ Shandong Key Laboratory of Innovation and Application Research in Basic Theory of Traditional Chinese Medicine, Shandong University of Traditional Chinese Medicine, Jinan, China; ^6^ Key Laboratory of Traditional Chinese Medicine Classical Theory, Ministry of Education, Shandong University of Traditional Chinese Medicine, Jinan, China

**Keywords:** ethnopharmacology, responsibility, 4R principles, animal ethics, animal research

## Abstract

Ethnopharmacology, a discipline focused on studying the medicinal use of natural materials by humans, plays a crucial role in addressing challenges in modern drug development. However, the traditional 3R principle—Replacement, Reduction, and Refinement—have limitations in guiding the ethical management of animal experimentation, conducting animal studies, and utilizing animal-derived materials in ethnopharmacological research. To address these gaps, the field has introduced the 4R principles, which expand the original framework by adding “Responsibility.” The Responsibility principle highlights the ethical obligation of researchers to consider the welfare of experimental animals during all procedures. It calls for researchers to take accountability for their actions and decisions, ensuring that they actively protect animal welfare and exhibit empathy across species. This principle reinforces the ethical foundation of ethnopharmacological research. To implement the 4R principles effectively, this article explores the dimensions of Reduction, Refinement, Replacement, and Responsibility in detail. For Reduction, strategies include minimizing animal use by developing optimized, efficient experimental designs, creating tissue banks to recycle animal samples, and improving success rates in animal modeling. These efforts collectively aim to enhance ethical standards while advancing scientific outcomes. In terms of Refinement, the goal is to minimize animal distress and pain by improving the experimental environment, refining operational procedures, ensuring strict control of experiments under anesthesia, and prioritizing non-invasive or minimally invasive techniques for data collection. For Replacement, the aim is to reduce the need for experimental animals by exploring alternative solutions. This includes substituting in vitro experiments for in vivo ones, using 3D organoids to replace animal organs, and applying deep learning technologies in ways that decrease animal use. The Responsibility principle focuses on enhancing researchers’ ethical obligations toward animal welfare. This can be achieved by improving regulations and policies governing animal experimentation, providing ethical training for technical personnel, and promoting awareness of animal welfare and ethical practices. The introduction and implementation of the 4R principles provide valuable guidance for the ethical conduct of animal experimentation in ethnopharmacological research, offering new insights and methodologies that support the responsible use of animals in scientific studies.

## Graphical Abstract

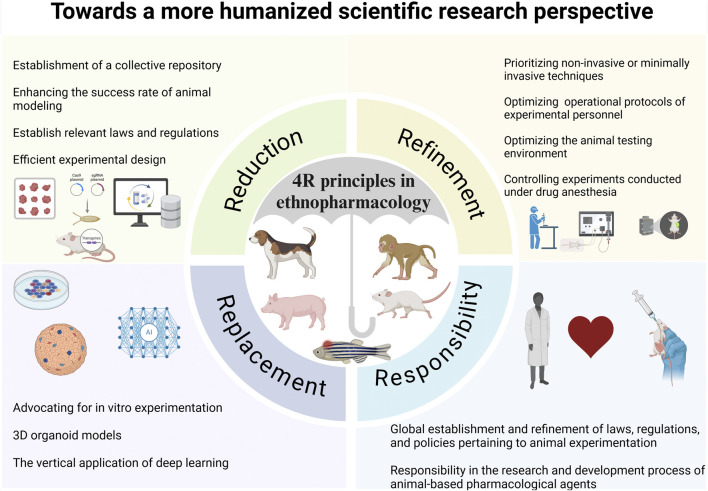

## 1 Introduction

Professor Bo Holmstedt initiated the ethnopharmacological study of active substances in exotic plants as early as 1967 (Atkinson, 2002). He defined ethnopharmacology as an interdisciplinary field focused on biologically active substances traditionally used or observed by humans. Its purpose is to preserve and document valuable cultural heritage that is at risk of being lost, while researching and evaluating these medicinal substances. Professor Holmstedt emphasized that the goal of studying traditional medicines is not to advocate for their return to original usage, nor to create traditional pharmacopeias (Bruhn and Rivier, 2019). Initially, ethnopharmacology sought to identify bioactive compounds in plants, fungi, animals, and minerals used in traditional medicine. However, it soon became clear that identifying bioactive compounds is only one aspect of the discipline’s broader scope (Reyes-García, 2010). Over time, ethnopharmacology has evolved into a field dedicated to exploring the medicinal potential of natural materials, including plants, fungi, microorganisms, animals, and minerals. As a challenging interdisciplinary research area, it is regarded by scholars as a bridge between the natural and medical sciences and the social and cultural sciences.

In this complex field, researchers have integrated methods from pharmacology, anthropology, and socio-cultural studies, leading to an organic fusion of these diverse approaches (Schultz and Garbe, 2023). Modern pharmacology typically aims to develop single active compounds with specific targets. While these highly effective drugs, including natural products and their derivatives, are widely used in clinical practice, they often fall short in treating complex human diseases such as cancer, diabetes, and malaria. In these cases, ethnopharmacology, particularly in the area of botany, plays a critical role (Li and Weng, 2017).

Mice, rats, dogs, and monkeys are commonly used in preclinical experiments due to their significant biological similarities to humans. In 2008, Katy Taylor analyzed data from countries that reported animal usage statistics to estimate global experimental animal use in 2005. Her survey revealed that laboratories in 37 countries utilized approximately 46.6 million animals. She then applied a statistical model to estimate usage in other regions, concluding that a conservative total of 58.3 million animals were used across 179 countries and regions. These animals were primarily used for tissue procurement, the breeding of genetically modified animals, and euthanasia due to surplus. However, the model likely underestimated the actual number of animals used (Taylor et al., 2008). A decade later, Taylor revisited this issue and estimated that the global number of animals used for scientific research had risen to 79.9 million in 2015, reflecting a modest increase of 36.9% (Taylor and Alvarez, 2019) compared to the 2005 estimate of 58.3 million (Bian et al., 2018). Despite their invaluable contributions to medical advancements, as highlighted by Thomas Hartung, over-reliance on animal models can introduce significant challenges in drug development (Hartung, 2024). Hartung noted that while animals like mice and rats have played a crucial role in improving human health, they cannot always reliably predict human outcomes, leading to potential setbacks in therapeutic progress.

The appropriate use of animal models is a critical aspect of the drug development process. Indian researcher Vidhya C. S. conducted a comprehensive survey on the use of various animal models, ranging from invertebrates to mammals, highlighting their roles in drug development and emphasizing the importance of animal welfare in experimentation (Vidhya et al., 2024). Cancer treatment provides a pertinent example. With hundreds of thousands of new cases diagnosed globally each year, the disease’s complexity poses significant challenges to medical advancements (Du et al., 2024). In 2024, Guo systematically reviewed the relationship between cancer treatment and preclinical animal models. In addition to the extensively used mouse models, the review examined the application of other animal models, including cats, beagles, monkeys, pigs, and rabbits. Guo categorized mouse models into five main types: (1) syngeneic and xenograft models, created through subcutaneous, intraperitoneal, intravenous, or intramuscular injections; (2) orthotopic models developed using chemical carcinogens and gene-editing techniques; (3) genetically engineered mouse models; (4) models induced by common heterocyclic aromatic carcinogens; and (5) models using nitrosamine carcinogens (Guo et al., 2024).

Reihaneh Alsadat Mahmoudian underscored the value of rodent models in cancer research, noting their highly similar genomic structures and physiological functions to humans. This similarity allows rodents, particularly mice and rats, to effectively simulate human cancer progression, making them indispensable in the development of anticancer drugs (Mahmoudian et al., 2023). The widespread use of experimental animal models, especially mice, has drawn significant attention to the ethical and moral issues associated with their use. These concerns emphasize the need for researchers to handle their interactions with experimental animals responsibly. For example, during the 1970s in China—a period marked by limited technological advancement and economic underdevelopment—scientists relied heavily on experimental animals in their exploration of artemisinin. Despite the ethical challenges, this effort ultimately led to the discovery of a life-saving compound that has benefited humanity. This historical example highlights the delicate balance between scientific progress and the ethical treatment of experimental animals.

## 2 Methodology

### 2.1 Search strategy

A three-tier search strategy was implemented: electronic databases (e.g., PubMed, Scopus, Web of Science), manual searching (relevant journals and conference proceedings), citation tracking (15 seminal papers identified in initial screening). We used a set of predefined search terms related to the 4R principles, ethnopharmacology, and ethical considerations in animal research. The search was limited to articles published in English to maintain consistency and accessibility for the intended audience.

### 2.2 Inclusion and exclusion criteria

The selection protocol required included studies to explicitly address the implementation of 4R principles and Responsibility within ethnopharmacological research contexts, particularly those involving animal-derived components in traditional medical systems. We prioritized peer-reviewed original research published between 2000 and 2024 that provided verifiable data on both ethical frameworks and practical application outcomes, while excluding non-empirical commentaries, theoretical discussions lacking case studies, and investigations limited to the conventional 3R paradigm without substantive integration of the Responsibility dimension. To ensure methodological rigor, studies were further excluded if they failed to document institutional ethics approval processes or omitted detailed descriptions of cultural adaptation strategies in traditional medicine contexts. Language accessibility considerations led to the exclusion of non-English publications lacking official translations, though we recognize this may introduce geographical bias in coverage.

## 3 Animal welfare global efforts and the “responsibility” principle

The ethical principles guiding animal experimentation are encapsulated in the “3Rs”: Replacement, Reduction, and Refinement. In ethnopharmacology, however, the unique nature of research often requires a significant number of experimental animals for drug development. This creates a compelling need to introduce a fourth “R” principle: Responsibility. While the “3Rs” address several aspects of animal welfare, they do not fully encompass the final frontier of animal protection. Adding the fourth “R”: Responsibility, addresses this gap by emphasizing the role of researchers in ensuring ethical practices. This principle highlights the importance of researchers exercising initiative, empathy, and a strong sense of responsibility in balancing scientific progress with respect for animal welfare. It integrates Responsibility into the “3R” framework, enhancing its effectiveness.

The “3Rs” were first introduced in the book Humane Experimental Technique (1959) by William M.S. Russell and Rex L. Burch of the University Federation for Animal Welfare (UFAW). Their framework aimed to improve the welfare of animals used in scientific experiments (“The Principles of Humane Experimental Technique,” 1960). Russell and Burch clearly outlined the sequential application of the 3R principle in animal experimentation: first, researchers should avoid using animals if non-animal methods can achieve the same scientific objectives; second, if animal experimentation is necessary, researchers must balance the number of animals used with the goals of the experiment, optimizing experimental design to minimize animal use; and third, the most crucial step, researchers must take all necessary measures to minimize the suffering of animals involved in research (Hubrecht and Carter, 2019). In scientific animal experimentation, the nature of several experiments inevitably cause both physical and psychological suffering of the animals. In some cases, completing the research may require sacrificing the animals. The introduction of the 3R principle has, to some extent, improved animal welfare and alleviated their suffering. As a result, these principles have been widely adopted by the global research community (Lewis, 2019).

Although current laws and regulations have not fully prohibited animal use in scientific research, these efforts have received widespread recognition worldwide. There is increasing awareness that the sacrifices made by animals for scientific progress should not be taken for granted. The contributions of experimental animals must be respected and their welfare carefully considered on a global scale. As discussed earlier, a complete ban on animal experimentation for advancing medical research is unrealistic. This holds true whether investigating the therapeutic mechanisms of ethnopharmacological remedies, as in the case of Tu Youyou’s malaria treatment, or conducting clinical trials of new chemical drugs. The crucial role of experimental animals in human development is undeniable. Researchers, who have direct contact with experimental animals, play a key role in this process. For them, the primary concern during experimentation is how to treat animals with compassion and respect while achieving valid research outcomes. To better protect animals used in research, the field of ethnopharmacology has refined the 3R principle by adding a fourth element - “Responsibility.” This has led to the development of the 4R principles, which have attracted significant attention from both researchers and society regarding the welfare of experimental animals. This paper, after reviewing new technologies and methods for optimizing animal model use and exploring alternatives, will further emphasize how researchers can improve the protection of experimental animals. The goal is to better integrate the 4R principles into ethnopharmacological research practices.

## 4 Optimizing the application of the reduction principle

### 4.1 Initiatives for advancing the optimization of efficient experimental design

A research project typically begins with careful experimental design. Professor Bryan Smucker has highlighted the importance of optimal experimental design, emphasizing that researchers should not simply adjust their experimental settings to fit traditional models but should instead adopt the best design for the specific scientific question at hand (Smucker et al., 2018). The role of interdisciplinary collaboration in this process is critical, as the complexity of scientific problems often demands deep analysis that can only be achieved through the exchange and integration of knowledge across different fields (Nguyen and Mougenot, 2022). Researchers, particularly those on the front lines, are the key implementers of scientific research. They play a crucial role in the experimental process. To ensure the design of scientifically sound and comprehensive experiments, researchers must undergo systematic training before initiating a study. For graduate students, a relevant assessment should be conducted prior to beginning experimental design to confirm that their knowledge is sufficient to apply the latest technologies. In the field of ethnopharmacology, for example, it is essential to explore cutting-edge technologies during the design phase to address challenges related to animal use in drug discovery, as well as to overcome issues such as high costs and long development cycles. Recent studies highlight the Virtu Dock DL deep learning pipeline, developed by the Vijil Chenthamarakshan team, as a significant advancement in accelerating the virtual screening process for drug discovery. This Python-based platform leverages deep learning technology, achieving an accuracy of 99% on the HER2 dataset, outperforming both Deep Chem (89% accuracy) and Auto Dock Vina (82% accuracy) (Noor et al., 2024).

In addition, the Rosetta VS structure-based virtual screening method, developed by the University of Washington research team, can accurately predict docking poses and binding affinity. The AI-driven virtual screening platform is capable of screening a billion-compound library in just 7 days, with identified hit compounds exhibiting binding affinities in the single-digit micromolar range (Zhou et al., 2024). The integration of computational technologies has notably improved experimental efficiency. Platforms like Virtu Dock DL and Rosetta VS not only achieve high accuracy rates in drug screening but also enable the rapid processing of large-scale compound libraries, surpassing traditional methods.

### 4.2 A collective repository for animal tissue sample recycling to foster a sharing culture

As early as 2015, researchers, including Marc van de Wetering, proposed creating a living organoid biobank by extracting tumor organoid cells from the adjacent normal tissue of colorectal cancer patients. This method has since been utilized in colorectal cancer research (van de Wetering et al., 2015). In the field of ethnopharmacology, the growing demand for experimental animals has led to the concept of constructing a collective tissue sample repository. The creation and global sharing of this repository could eliminate the barriers to scientific exchange caused by economic, political, and cultural differences. By optimizing the use of animal sample resources, it would help reduce unnecessary animal sacrifice. Integrating shared animal experimental models, including those for diseases and their complications, allow researchers across different fields to collaborate by sharing animal tissue resources for pharmacological studies. Further mechanistic investigations can be conducted using techniques such as cell cultures or organoids. To enhance the effectiveness of this repository, we propose developing a database with comprehensive sample information, including source, type, processing methods, and preservation status.

### 4.3 Strategies for enhancing animal modeling success rates

In 2012, Nobel laureates Charpentier and Doudna first described the clustered regularly interspaced short palindromic repeats (CRISPR) associated endonuclease protein 9 (Cas9). The technology has been applied to a variety of animal models, including mice, rats, pigs, monkeys, and fish. Moreover, CRISPR/Cas9-based cellular models have reduced the dependence on animal models to some extent. For example, the traditional Chinese herbal medicine *Melia toosendan* Sieb (Chuanlianzi), which has been used for approximately 1,500 years to treat abdominal pain and gastrointestinal parasites, has also been studied using CRISPR/Cas9 technology. Recent studies have shown that the tetra-cyclic triterpenoid compound *toosendanin* (TSN), purified from Chuanlianzi, demonstrates potential anti-proliferative, autophagy-inhibitory, and pro-apoptotic effects in various human cancers (Dong et al., 2022; Kai et al., 2018; Zhang et al., 2005). However, its precise anti-cancer mechanisms and drug targets remain unclear. CRISPR-Cas9 knockout technology offers a powerful tool for identifying key genes associated with drug sensitivity. Additionally, studies indicate that high-throughput CRISPR-Cas9 library genome screening can aid in selecting effective anti-cancer drugs (Chan et al., 2022). For instance, Yang et al. used a CRISPR-Cas9 library to screen for key genes targeted by TSN in hepatocellular carcinoma cells. Researchers transfected a whole-genome CRISPR-Cas9 knockout library (targeting nearly 20,000 human genes) into the 97L hepatocellular carcinoma cell line. After exposing these cells to TSN and culturing them for 7 days, they performed positive and negative selection. Gene Ontology (GO) enrichment analysis revealed that the significantly downregulated genes were primarily involved in ribosome biogenesis-related pathways. This analysis ultimately identified a gene containing the WW-domain containing oxidoreductase (WWOX) domain as a drug target for TSN’s anti-tumor effects, a finding that was further validated in subsequent experiments (Yang et al., 2021).

However, CRISPR-Cas9 technology has limitations, including the risk of off-target effects, which can lead to serious consequences (Li et al., 2023). To address this, Dmitrii et al. developed engineered PsCas9 (ePsCas9), which enhances target activity while minimizing off-target effects (Degtev et al., 2024), thereby offering a safer alternative. The use of transgenic technology in generating model animals has helped alleviate the suffering associated with traditional surgical modeling methods. The CRISPR-Cas9 gene editing system, a major breakthrough in molecular biology, plays a critical role in this advancement. However, the technology also presents several challenges, particularly in the creation of transgenic animals. The breeding process often results in a surplus of non-phenotypic heterozygotes and animals carrying non-target genes, several of which are euthanized. The unnecessary birth and subsequent termination of these animals raise significant ethical concerns, demanding that researchers further improve technology and refine techniques to minimize off-target effects and increase the proportion of homozygous animals.

## 5 Optimizing the application of the refinement principle

### 5.1 Further optimization of animal experimentation environments and operational protocols to reduce animal distress

The guide establishes standards for facility construction, housing, husbandry, and care (including space allocation), which are critical for animal welfare. Standardized operating procedures (SOPs) are essential for experimental operators to minimize distress caused by mishandling. Researchers must address animal discomfort and anxiety by employing positive reinforcement training to reduce fear and stress. Building on the work of Jane L. Hurst and Rebecca S. West, who in 2010 emphasized the aversive effects of the tail-pinch method, innovative approaches such as tunnels or open-hand techniques should be adopted to promote voluntary approach behavior in mice (Hurst and West, 2010). In pharmacological experiments, one challenge is to accurately simulate human drug administration while ensuring animal welfare. The advent of Micropipette-guided Drug Administration (MDA), a non-invasive drug delivery method, addresses this issue. MDA allows precise control over timing and dosage while safeguarding animal welfare. In 2020, Joseph Scarborough and colleagues developed this technique and evaluated its applicability and efficacy in a murine model of maternal immune activation (Scarborough et al., 2020). In 2023, Marie Heraudeau and others demonstrated, for the first time, that MDA is also applicable in rats (Heraudeau et al., 2023). In pharmacological experiments involving large animals, such as dogs, pigs, and non-human primates, the complex tasks of moving, restraining, and administering drugs pose significant challenges. The use of force to achieve experimental goals conflicts with the fundamental principles of animal ethics. To address this, Positive Reinforcement Training (PRT) has emerged as an effective solution. Introduced by psychologist B. F. Skinner, PRT is based on operant conditioning, a learning theory in which behavior is influenced by its consequences. In this approach, specific behaviors are reinforced by rewarding stimuli, such as food, thereby increasing the likelihood of those behaviors recurring in the future (McDouall, 2021). In 2010, Gail Laule investigated the application of PRT in large animal experiments. Laule demonstrated that PRT not only enhances animal cooperation during experimental procedures, reduces harm, and improves efficiency, but also fosters a harmonious balance between humans and animals, making it an ideal solution for such studies (“Positive Reinforcement Training for Laboratory Animals - The UFAW Handbook on the Care and Management of Laboratory and Other Research Animals - Wiley Online Library,” n. d.). In 2012, Jaine E. Perlman provided a comprehensive analysis of positive reinforcement training for non-human primates. Despite scientific evidence showing that animal training can effectively reduce fear and other negative emotions, these methods have not been widely incorporated into animal care and management plans for experimentation. Perlman advocated for the integration of such training into animal husbandry and experimental procedures (Perlman et al., 2012). In the 1990s, Karen and Gary Wilkes developed Clicker Training (CT), initially used for canine training, which was later adapted for use in experimental animal research. More recently, in 2023, researchers successfully applied clicker training, luring, and positive reinforcement techniques to facilitate behavioral testing in pigs, a large animal species, without eliciting fear or anxiety (Jønholt et al., 2021). This highlights the importance of training animals before experimentation to improve their ability to cope with the procedures. Pre-experimental training is critical for maintaining appropriate stress levels in animals. During the preparation phase, regular care for experimental animals, such as mice, through feeding and gentle handling, not only minimizes harm during experiments but also reduces stress and prevents bias in experimental data.

### 5.2 Stringent control of drug anesthesia in experimental procedures to alleviate animal pain perception

In pharmacological research, invasive procedures require the use of appropriate anesthetics to ensure animal welfare. Anesthesia is essential for immobilizing animals during experiments, selecting the correct anesthetic depth, and administering analgesics to alleviate suffering. It also ensures the safety of both animals and experimenters. However, the choice of anesthetic is crucial, as it can influence the accuracy of experimental outcomes. For instance, Li Ru’s study shows that different anesthetics can produce distinct effects in mice, with sevoflurane potentially enhancing tumor cell lung metastasis *via* the IL6/JAK/STAT3 pathway (Li et al., 2020). In 2024, Sang Su Oh and Heather L. Narver provided a comprehensive review of anesthesia and analgesia in mice and rats, highlighting significant advancements in anesthesia techniques. These advancements have moved away from a “one-size-fits-all” approach, favoring customized anesthesia protocols. Furthermore, the management of analgesia during experimental procedures has become more specialized (Oh and Narver, 2024). As social systems progress, there is hope for the establishment of global, unified standards for anesthetic use, which would guide researchers in selecting the most appropriate anesthetics based on specific experimental needs.

### 5.3 Prioritizing non-invasive or minimally invasive techniques and least harmful experimental methods for data collection

Technological advancements have increasingly emphasized the development of non-invasive or minimally invasive techniques in ethnopharmacology, particularly for drug development, to enable data collection without causing harm to experimental animals. In cognitive neuroscience, experimental paradigms have shifted towards more naturalistic approaches. Researchers at the Wu Tsai Neurosciences Institute at Stanford University have recently developed a non-invasive method for remotely controlling targeted brain circuits in animals engaged in natural behaviors. This innovation addresses a critical need in neuroscience by enabling researchers to investigate the roles of specific neurons and circuits in the brain during normal activities, such as social interactions in mice (Wu et al., 2022). This breakthrough suggests that invasive methods, such as implanting stimulation electrodes, optical fibers (Acarón Ledesma et al., 2019; Hong et al., 2017; Hong and Lieber, 2019), and chronic brain implants, which cause permanent tissue damage, skull injury, and chronic immune responses at the implant site, may soon become obsolete (Salatino et al., 2017). The introduction of this technology represents a significant step forward for drug development in cognitive neuroscience.

In 2024, Yu Yang provided a comprehensive review of non-invasive physiological sensing technologies in modern medicine, highlighting eight techniques: photoplethysmography (PPG), electroencephalography (EEG), electromyography (EMG), electrocardiography (ECG), computed tomography (CT), magnetic resonance imaging (MRI), ultrasonography (US), and electrical impedance tomography (EIT). These technologies can be fully applied to animal experiments, offering viable alternatives to reduce harm to animals (Yu et al., 2024). Their immediate implementation in animal research has the potential to replace invasive procedures and significantly improve ethical standards in preclinical studies. At the 2023 ICMMT, Zhejiang University’s School of Electronics and Information Engineering introduced a novel, rapid non-invasive detection method for monitoring vital signs and the autonomic nervous system in mice. This innovation highlights the integration of advanced electronic technologies with life sciences, illustrating a growing interdisciplinary trend (Ye et al., 2023).

## 6 Optimizing the implementation of the replacement principle in animal research ethics

### 6.1 *In vitro* experiments as a substitute for *in vivo* procedures: Ensuring the welfare of laboratory animals

The shift from the 20th-century research paradigm, which heavily relied on animal experimentation, to a 21st-century approach prioritizing *in vitro* methods marks a significant advancement in societal concern for animal welfare. In ethnopharmacology, the discovery of artemisinin serves as a key example of this transition. Initially, animal models played a central role in the discovery process, allowing subsequent researchers to employ modern techniques for anti-malarial drug screening. This highlights the foundational role of animal experiments in developing targeted therapies derived from traditional remedies. Experimental animals were essential in the discovery of artemisinin, particularly given the complexity of *Artemisia annua*’s components and the limited technological resources available at the time. The pivotal breakthrough occurred on 4 October 1971, when sample 191, an ethyl ether extract of *Artemisia annua*, was identified, demonstrating complete inhibition of malaria in both rodents and primates (Tu, 2016). By 2024, the intersection of scientific advancement and animal welfare has led emerging researchers to favor *in vitro* methods, reducing the reliance on animal testing.

The “World Malaria Report 2022″confirms the ongoing global malaria burden through 2021, with artemisinin-based combination therapies (ACTs) remains the treatment of choice for severe malaria. China’s 2021 designation as malaria-free by the WHO highlights the success of ACTs (“World malaria report 2022,” n. d.). This achievement is attributed to *Artemisia annua* (Zheng et al., 2020). Researchers from the University of California and Yale University developed MED6-189, a compound effective against both susceptible and resistant *Plasmodium falciparum* strains *in vitro* and in humanized mouse models. The design of MED6-189 is based on isocyanoterpene (ICT), a class of marine sponge-derived natural products known for their robust antibacterial, antifungal, and antimalarial properties, particularly kalihinol derivatives. Overcoming challenges related to the scalability of natural products and understanding their mechanisms of action, the team successfully synthesized MED6-189 and related derivatives (Chahine et al., 2024).

The ethnopharmacological discovery of artemisinin highlights the complexity of experimental design. Tu Youyou’s team initially screened over 2,000 herbal preparations, selecting 640 compounds with potential antimalarial activity. They then extracted over 380 compounds for testing in murine malaria models (Tu, 2011). The critical role of animal models in advancing human health is underscored by the validation of MED6-189s efficacy against *P. falciparum* in mice and *P. knowlesi* in monkeys. Clinical application of MED6-189 will require additional, complex studies across various animal models. While experimental animals remain essential for health advancements and ethnopharmacology, emerging scientific methods and ethical considerations emphasize the need to balance the interests of humans and animals. *In vitro* cell cultures are increasingly central to drug discovery, and innovative alternatives—such as the EU-funded method using human blood cells to detect pyrogens—are gaining prominence. This method has the potential to significantly reduce animal use in over 200 laboratories worldwide (“EU project develops alternative to animal experiments,” n.d.).

### 6.2 Utilization of 3D organoids to reduce animal organ demands in research

In ethnopharmacology, researchers are actively seeking high-quality alternatives to experimental animals. The use of organoid technology has significantly reduced reliance on animal models, addressing interspecies differences and providing a more accurate simulation of human physiology (Bian et al., 2018). Professor Jean-Michel Besnier of Sorbonne University argues that societal progress requires recognizing animals as sentient beings with emotions and consciousness, rather than as disposable subjects for experimentation. Emerging insights from animal behavior and psychology, including the concept of “animal culture,” are reshaping attitudes toward animal testing. The UniMat organoid culture platform, developed by Dohui Kim, has transformed organoid production by addressing the limitations of traditional methods. This platform utilizes a three-dimensional geometrically designed permeable membrane to facilitate the exchange of soluble factors, integrating this innovation into cell culture systems to produce uniform, large-scale mature organoids. Its efficacy has been demonstrated in the successful large-scale production of kidney organoids derived from human-induced pluripotent stem cells (Kim et al., 2024). In 2013, Jürgen Knoblich from the Institute of Molecular Biotechnology at the Austrian Academy of Sciences, along with developmental biologist Madeline Lancaster from the University of Cambridge, cultivated the first three-dimensional brain organoids from human pluripotent stem cells. This research utilized biomaterial matrigel to simulate brain tissue and a rotating bioreactor to enhance nutrient absorption and oxygen diffusion. The resulting brain organoid cultures, which contained multiple brain regions, were developed through continuous three-dimensional suspension culture and the addition of growth factors that promoted neural development (Lancaster et al., 2013). More recently, a team led by Professor Xiangyang Fei (Shanghai Tech University, China) developed a brain organoid with neural projections that closely mimic *in vivo* models. This advancement holds promise for drug evaluation and assessment, particularly for research involving the spinal trigeminal nucleus (Pang et al., 2024). Ongoing innovation and development in brain organoid technology have significantly advanced the understanding and treatment of complex brain diseases, contributing to human health while minimizing the need for experimental animals.

### 6.3 Vertical applications of deep learning enhance the reliability of predictive computations, reducing experimental need

In traditional Chinese medicine, the vast array of natural molecular compounds presents a challenge comparable to finding a needle in a haystack. However, technological advancements, particularly in artificial intelligence (AI), have provided researchers with powerful tools for discovering new drugs. AI applications, such as virtual screening and computational design, have significantly improved the accuracy of identifying promising natural molecules (Lin et al., 2022). In 2024, the Nobel Prizes in Chemistry and Physics recognized significant advancements in AI (“The Nobel Prize in Chemistry 2024,” 2024). The Chemistry Prize was awarded jointly: David Baker from the University of Washington received half for his groundbreaking work in designing novel proteins, while Demis Hassabis and John M. Jumper of Google DeepMind in the United Kingdom received the other half for developing the AlphaFold2 model. Released on 15 July 2021, AlphaFold2 solved the 50-year-old “protein folding problem,” enabling the prediction of complex protein structures (Service, 2020). This model has predicted over 200 million protein structures and is now used by over 2 million researchers in more than 190 countries. Its technological breakthrough lies in the ability to predict the three-dimensional structures of proteins based on their amino acid sequences, which is essential for understanding protein function and advancing drug development (Jumper et al., 2021). On July 22, the team used AlphaFold2 to predict the structure of the human proteome, covering nearly 60% of the amino acid positions with high-confidence predictions (Tunyasuvunakool et al., 2021). On 8 May 2024, the teams of John Jumper at DeepMind and Demis Hassabis at Isomorphic Labs launched AlphaFold3, an upgraded version of the AI model that can predict protein interactions with other biomolecules with unprecedented accuracy. The new model surpasses previous prediction tools, including AlphaFold-Multimer, in its ability to predict the structure of almost all types of complexes within protein databases (Abramson et al., 2024). By parsing the complex interactions between proteins and other molecules, AlphaFold3 deepens our understanding of biological processes and is poised to transform drug discovery, fueling the emerging industry of AI-assisted pharmaceuticals.

## 7 Proposing the principle of responsibility: Balancing scientific development with animal welfare through a human-centered approach

### 7.1 Global refinement of regulations and policies relating to animal experiments in ethnopharmacological research

In ethnopharmacology, animal models remain essential for certain experimental studies; however, the efficiency of translating these research findings into clinical applications has been inadequate. Increasing concerns about animal welfare, along with corresponding regulations, are driving researchers to reduce animal use. As a result, balancing the ethical considerations of animal welfare with research objectives has become critically important (Budolfson et al., 2023). In 2023, the European Commission proposed a ban on the caging of farm animals, underscoring its commitment to prioritizing animal welfare throughout the “farm to fork” process (Duval and Lecorps, 2023). In recent years, public concern for animal welfare has also risen significantly in China. In 2012, Meng Xiuxiang highlighted that China’s approach to animal welfare is in a transitional phase (Meng et al., 2012). Although the country has yet to pass national legislation specifically addressing animal welfare, the legislative process is influenced by multiple factors, particularly economic considerations, as the implementation of animal welfare standards could negatively impact production activities. The paper emphasizes that collaboration between Chinese researchers, non-governmental organizations, and the international academic community will be crucial for integrating effective global policies into the development of Chinese standards. Li (2022) examines the progress and challenges of animal welfare in China, noting that although the country still has considerable ground to cover, increasing public awareness and sustained efforts signal a positive trend. Peter J. Li (Granger, 2022) explores how traditional culture shapes Chinese attitudes toward animals and identifies gaps in China’s political and legal systems concerning animal welfare. He also discusses the various animal welfare crises faced by the country and provides a series of recommendations to promote improvements, despite the current challenges. While acknowledging global variations in circumstances, this paper advocates for the establishment of unified global standards and the implementation of consistent laws and regulations to guide the practices of researchers worldwide, particularly in relation to the 3R principle.

### 7.2 A unique perspective of ethnopharmacology: Embracing responsibility in the research and development process of animal-derived medicines

In ethnopharmacological research, certain animal species are not only used as models but are also directly employed as medicinal materials—an aspect that often receives less attention than the welfare of animal models (Etkin, 1988). The traditional Chinese medicinal use of hedgehog skin for the treatment of hematochezia and hemorrhoids has been documented (Xiahou et al., 2022). In the realm of traditional Chinese medicine, bear bile powder has been utilized as a preventive measure against the Hepatitis C virus (Wang et al., 2013)While much of the focus in pharmacology has been on plant-based medicines, animals play a significant role in the pharmacopeia. According to WHO data, 8.7% of essential chemicals are animal derived. This is evident globally, from prescription drugs in the United States to ethnomedicines in India, and in systems like Unani and Brazilian folk medicine, which rely extensively on animal-based substances to treat parasitic infections and systemic diseases (Ahmad et al., 2023; Alves, 2009; Mutua et al., 2020). A 2024 study on the Gelao community in northern Guizhou, China, meticulously documented ethnozoological medicinal practices, reviewing 55 animal-based remedies. Notably, the community’s use of animals for medicinal purposes is accompanied by conservation efforts, including bans on hunting during breeding and lactation, restrictions on killing immature animals, and strict prohibitions on using rare and endangered species. Most medicinal ingredients are sourced from domesticated or commercially available animals (Liu et al., 2024).

Before modern civilization, humans interacted with animals for self-protection, and the introduction of each traditional animal-based medicinal material involved extensive trial and error. Modern medicine now uses advanced technologies to find animal-derived substitutes in an effort to reduce harm to animals. Examples include synthetic musk (Lv et al., 2022) and artificial bear bile. Historically, bear bile has been a valuable and effective material in traditional Chinese medicine, but the practice of extracting bile from captive bears raises significant ethical concerns regarding animal welfare. Professor Yushi Shan’s team has developed an artificial substitute for bear bile, which was created in collaboration with the Institute of Materia Medica, Chinese Academy of Medical Sciences, and Zhongshan Anshi Biopharmaceutical Co., Ltd. This substitute received national clinical approval in 2018. Phase I clinical trials demonstrated good safety, and Phase II trials are currently underway at more than 20 hospitals across China (“Artificial Bear Bile,” 2024).

Additionally, the team has pioneered a new model for studying the active substances in endangered animal-based medicines. They have successfully elucidated the mechanisms of bear bile, antelope horn, and pangolin scales in treating diseases, while creating artificial substitutes with efficacy equivalent to the original materials, such as artificial bear bile powder, biomimetic antelope horn powder, and biomimetic pangolin scale powder. This work has addressed international challenges related to the structure, function, and production of keratin and revealed its significant antipyretic effects, among other properties. These findings break through previous limitations in understanding keratin’s pharmacological effects, offering substantial scientific and economic value. The team has also established a novel approach for creating substitutes for endangered animal-based medicines using cell factory technology, positioning themselves as leaders in this field. In the context of ethnopharmacology, natural materials are used as medicinal substances, raising important considerations regarding animal welfare. These concerns are particularly relevant within the framework of the 3R principle (Replacement, Reduction, Refinement) established by modern pharmacology. How to improve animal welfare, along with the corresponding ethical reviews and regulatory measures for animals used in ethnopharmacological research, remains a key topic for ongoing academic discussion.

## 8 Discussion

This article explores the significance of the 4R principles in ethnopharmacology, focusing on their application in specific experiments. It provides an updated perspective on the traditional 3R principle, introducing a fourth “R” – Responsibility - within the context of ethnopharmacology, building on the established framework of Replacement, Reduction, and Refinement. While the traditional 3Rs have significantly advanced efforts to reduce animal use, improve experimental methodologies, and enhance animal welfare, they remain insufficient to address the evolving demands of scientific research. Therefore, the addition of the fourth “R,” Responsibility, aims to further elevate the ethical standards in animal research, ensuring that animal welfare is comprehensively considered throughout the experimental process.

Responsibility, as an essential complement to the 3R principle, plays a crucial role in scientific research and must not be overlooked. First, Responsibility supports Replacement, which involves identifying alternative methods to reduce the need for experimental animals. For example, advances in cell culture techniques and organoid models offer promising alternatives to animal experiments. However, these technologies may still rely on animal-derived materials, such as fetal bovine serum. In such cases, researchers are responsible for ensuring that the procurement of these materials adheres to the highest ethical standards and, where possible, seeks non-animal alternatives. Second, Responsibility is integral to Reduction, which emphasizes minimizing animal use in experimental design. This principle not only involves reducing the number of animals used but also optimizing experimental designs to avoid unnecessary repetition and improve efficiency. Lastly, Responsibility underpins Refinement, which focuses on improving experimental conditions to reduce animal suffering. Researchers must prioritize enhancing the wellbeing of animals during the research process by refining methods to minimize discomfort and distress. This includes providing a proper living environment and meticulous care and monitoring throughout the experiment to ensure the animals’ health and comfort. In practice, the Responsibility principle highlights the critical role of researchers as the primary executors of experiments. Researchers are not only responsible for designing and conducting experiments but also for serving as the primary stewards of the animals involved. They must acknowledge the sentience of experimental animals and build a positive rapport with them to ensure their wellbeing throughout the process. Going forward, the focus is on ensuring that researchers approach the animals in their experiments with seriousness and compassion, maintaining deep respect for their welfare. This responsibility entails treating experimental animals with empathy and reverence. Moreover, for animals used as donors, such as genetically modified pig hearts transplanted into humans (Mohiuddin et al., 2023), researchers must take on the responsibility of safeguarding these animals and ensuring that their treatment adheres to ethical standards. Although fetal bovine serum is widely used in cell and organoid cultures, and its procurement follows strict ethical and animal welfare guidelines, it still requires researchers to reflect on whether this practice genuinely aligns with animal welfare principles. With the global commercial cattle population estimated at two billion, the question arises whether current alternatives can effectively replace animal-based practices. These considerations call for the application of the Responsibility principle, encouraging researchers to limit animal use and minimize potentially harmful procedures, while still meeting experimental requirements. While the traditional 3R principle primarily focus on technical improvements, they overlook the responsibility of researchers as the direct executors of experiments. In contrast, the Responsibility principle places researchers at the center, emphasizing the impact of their decisions and actions on animal welfare. As the leaders of their projects, researchers wield significant power, with the design and execution of experiments resting entirely within their discretion. Thus, the introduction of the Responsibility principle highlights the human factor behind the traditional 3R principle, prompting each researcher to reflect on their conscience and sense of duty. By focusing on the individual—the decision-maker and executor—true respect for and protection of animal welfare can be achieved, grounded in the Responsibility principle.

Additionally, the influence of religious beliefs, cultural backgrounds, and political systems on animal experimentation must be considered. Ethics can vary significantly across different cultural contexts, and these differences affect the selection of animals for experimental research. Recognizing such cultural sensitivities, ethnopharmacology has incorporated the Responsibility principle as a key supplement to the traditional 3R principle. Although promoting unified standardized management is challenging due to diverse cultural and political contexts, it is essential to develop approaches to animal experimentation that align with the 4R principles while ensuring the welfare of the animals involved in research. The ethical review of animal experiments must go beyond ethical principles and include an evaluation of the experimental design, weighing the scientific benefits against the costs of laboratory resources. While efforts to minimize harm, such as the use of computer models, tissue cultures, or cell cultures as alternatives to animal testing, are common, public concerns about animal experimentation persist. Research that does not adhere to ethical standards may face significant scrutiny and criticism. This raises the question of whether the 4R principles in ethnopharmacology are sufficient to meet current experimental needs. Furthermore, we must consider whether an additional R, Refusal, should be introduced, whereby animal experiment proposals that do not conform to the 4R principles are not approved. Whether based on the 3R or 4R framework, research involving animals increasingly reflects humanistic concerns. Improving this process will continue to be an ongoing challenge.

## 9 Conclusion

In conclusion, the integration of the Responsibility principle into the 3R framework represents a critical evolution in ethnopharmacology’s ethical paradigm, emphasizing researcher accountability as the keystone of animal welfare. While this fourth R strengthens decision-makers’ capacity to balance scientific progress with ethical imperatives, its global implementation faces multilayered challenges. Cross-border disparities in regulatory rigor, resource allocation, and cultural valuations of animal life create friction in harmonizing 4R adoption. Collaborative frameworks must bridge these divides by engaging policymakers, international bodies (e.g., ICLAS, OIE), and local stakeholders to develop context-sensitive guidelines that respect cultural diversity while upholding core welfare standards. For instance, industrialized nations’ advanced infrastructure contrasts sharply with resource-limited regions where basic Refinement protocols remain aspirational, necessitating tiered implementation strategies and technology-sharing initiatives. Simultaneously, divergent scientific traditions—from Western evidence-based models to Indigenous knowledge systems—require dialogue to align ethical practices without erasing epistemological diversity. The Responsibility principle must evolve beyond individual researchers to encompass institutional and transnational accountability, fostering partnerships that address supply-chain ethics, training inequities, and inconsistent enforcement. Crucially, this demands standardized metrics for auditing 4R compliance across jurisdictions while maintaining flexibility for region-specific adaptations. Ethnopharmacology’s global nature positions it uniquely to model such cooperation, but success hinges on reconciling universal ethics with localized realities—a task as complex as the field’s multicultural foundations. Only through sustained interdisciplinary and international commitment can the 4R framework transcend aspirational rhetoric to drive measurable, equitable advancements in animal welfare.
